# A Hepatic Protein, Fetuin-A, Occupies a Protective Role in Lethal Systemic Inflammation

**DOI:** 10.1371/journal.pone.0016945

**Published:** 2011-02-08

**Authors:** Wei Li, Shu Zhu, Jianhua Li, Yan Huang, Zhou Rongrong, Xuegong Fan, Huan Yang, Xing Gong, N. Tony Eissa, Willi Jahnen-Dechent, Ping Wang, Kevin J. Tracey, Andrew E. Sama, Haichao Wang

**Affiliations:** 1 The Feinstein Institute for Medical Research, Manhasset, New York, United States of America; 2 Department of Emergency Medicine, North Shore University Hospital, Manhasset, New York, United States of America; 3 Department of Infectious Diseases, Xiangya Hospital, Central South University, Changsha, People's Republic of China; 4 Pulmonary Critical Care and Sleep Medicine Section, Baylor College of Medicine, Houston, Texas, United States of America; 5 Department of Biomedical Engineering, Biointerface Laboratory, RWTH Aachen University Hospital, Aachen, Germany; Louisiana State University, United States of America

## Abstract

**Background:**

A liver-derived protein, fetuin-A, was first purified from calf fetal serum in 1944, but its potential role in lethal systemic inflammation was previously unknown. This study aims to delineate the molecular mechanisms underlying the regulation of hepatic fetuin-A expression during lethal systemic inflammation (LSI), and investigated whether alterations of fetuin-A levels affect animal survival, and influence systemic accumulation of a late mediator, HMGB1.

**Methods and Findings:**

LSI was induced by endotoxemia or cecal ligation and puncture (CLP) in fetuin-A knock-out or wild-type mice, and animal survival rates were compared. Murine peritoneal macrophages were challenged with exogenous (endotoxin) or endogenous (IFN-γ) stimuli in the absence or presence of fetuin-A, and HMGB1 expression and release was assessed. Circulating fetuin-A levels were decreased in a time-dependent manner, starting between 26 h, reaching a nadir around 24–48 h, and returning towards base-line approximately 72 h post onset of endotoxemia or sepsis. These dynamic changes were mirrored by an early cytokine IFN-γ-mediated inhibition (up to 50–70%) of hepatic fetuin-A expression. Disruption of fetuin-A expression rendered animals more susceptible to LSI, whereas supplementation of fetuin-A (20–100 mg/kg) dose-dependently increased animal survival rates. The protection was associated with a significant reduction in systemic HMGB1 accumulation *in vivo*, and parallel inhibition of IFN-γ- or LPS-induced HMGB1 release *in vitro*.

**Conclusions:**

These experimental data suggest that fetuin-A is protective against lethal systemic inflammation partly by inhibiting active HMGB1 release.

## Introduction

Sepsis refers to a systemic inflammatory response syndrome resulting from a microbial infection, and is partly propagated by innate immune cells such as macrophages. Macrophages are equipped with pattern recognition receptors (such as TLR2, TLR4, and TLR9) [1]–[3], and can recognize pathogen-associated molecular patterns (PAMPs, such as endotoxin) [3]–[6], as well as damage-associated molecular patterns (DAMPs, such as HMGB1) [3], [4], [7], [8]. In response to various PAMPs or DAMPs, innate immune cells release many cytokines (such as TNF-α, IL-1, or IFN-γ) to orchestrate an inflammatory response [9]. Although an appropriate response is required to defend against infection, an uncontrolled systemic inflammation may adversely contribute to the pathogenesis of sepsis.

We discovered that HMGB1 is released by activated macrophages [5], and contributes to the pathogenesis of sepsis [10]. Like other danger signal molecules (such as heat shock proteins) [11], [12], extracellular HMGB1 functions as an alarmin signal to recruit, alert, and activate innate immune cells [10], [13]. For instance, HMGB1 can activate immune cells to produce various cytokines and chemokines [7], [8], [14]–[16], thereby sustaining a potentially injurious inflammatory response in sepsis [10], [13]. Consistently, anti-HMGB1 antibodies [5], [17] or inhibitors (e.g., tanshinones, ethyl pyruvate, nicotine, stearoyl lysophosphatidylcholine, or epigallocatechin-3-gallate) [5], [18]–[24] confer protection in animal models of endotoxemia and sepsis.

The liver orchestrates a host defense response by altering (re-prioritizing) the synthesis and systemic release of “acute phase proteins” (APPs, such as fetuin-A, also termed the alpha-2-HS-glycoprotein for the human homologue) [25]. The expression of fetuin-A is counter-regulated by proinflammatory cytokines such as TNF-α, IL-1, and IL-6 [26], classifying it as a negative APP [27], [28]. However, plasma fetuin-A levels were elevated in patients after ischemic stroke [29], [30] or cattle after trauma [31], implying that fetuin-A may also function as a positive APP. A wide range of biological functions have been proposed for fetuin-A based on either its structural similarities to other proteins, or interaction with biogenic molecules. For instance, fetuin-A shares sequence similarity to insulin receptor tyrosine kinase [32], [33] and type II TGF-β receptor [34], and has thus been proposed as an inhibitor of insulin or TGF-βsignaling pathways. As a glycoprotein, fetuin-A carries two N-linked and three O-linked oligosaccharide chains that terminate with sialic acid residues, and can bind biogenic cationic ions (e.g., Ca^2+^) and other anti-inflammatory molecules (e.g., spermine) [35], [36]. Accordingly, fetuin-A has been proposed as an endogenous inhibitor of pathological mineralization/calcification [37]–[40], and an opsonin of cationic molecules (such as spermine) [36].

At extremely high concentrations (e.g., 3500 µg/ml), crude bovine fetuin-A preparation (purity >98%, Sigma-Aldrich) abolishes LPS (10 µg/ml)-induced release of nitric oxide and IL-1β in macrophage cultures [41]. In animal models of carrageenan-induced paw edema or cerebral ischemia, administration of fetuin-A merely attenuated early, but not late, inflammatory response in the paw [42] or ischemic brain [43]. It was previously unknown whether: i) fetuin-A functions as a negative or positive APP in lethal systemic inflammation (LSI), ii) other early proinflammatory cytokines also counter-regulate hepatic fetuin-A expression, iii) fetuin-A confers a long-lasting protection against LSI by inhibiting late proinflammatory mediators. Here we showed that fetuin-A functions as a negative APP, and confers protection against LSI partly by attenuating HMGB1 release.

## Results

### Circulating fetuin-A levels were temporally reduced in lethal endotoxemia and sepsis

**Figure 1:**
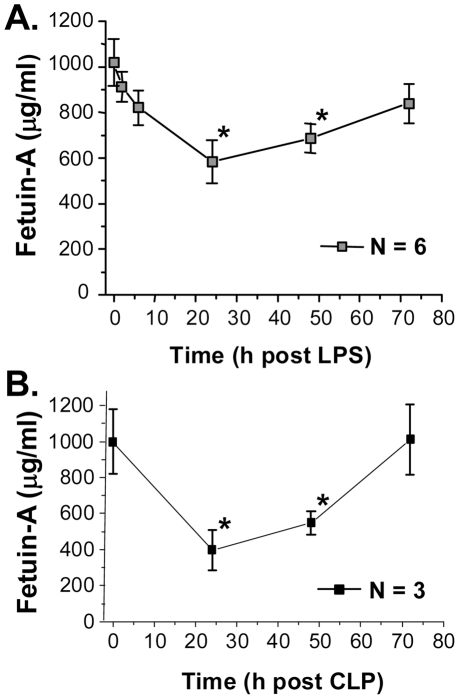
Circulating fetuin-A levels were temporally decreased during endotoxemia and sepsis. Balb/C mice were subjected to endotoxemia (LPS, 10 mg/kg, i.p.) or sepsis (induced by CLP), and sacrificed at indicated time points to collect blood. Serum fetuin-A levels were determined by Western blotting analysis with reference to standard curve generated with purified fetuin-A at various dilutions, and expressed as mean ± SD of 3–6 independent experiments (N = 3–6), with 4–6 animals included in each group. *, *P*<0.05 versus “0 h post LPS” (**Panel A**) or “0 h post CLP” (**Panel B**).

To understand the role of fetuin-A in LSI, we measured its circulating levels in murine models of lethal endotoxemia and sepsis. Circulating fetuin-A levels were decreased in both endotoxemic (**Fig. 1A**) and septic (**Fig. 1B**) mice in a time-dependent fashion, with maximal reduction (by 50–60%) 24–48 h after onset of these diseases. Afterwards, fetuin-A levels started to increase, returning towards basal levels approximately 72 h post endotoxemia (**Fig. 1A**) or sepsis (**Fig. 1B**), supporting the notion that fetuin-A functions as a negative APP in murine models of LSI.

### Role of early proinflammatory cytokines in the regulation of hepatic fetuin-A expression

**Figure 2:**
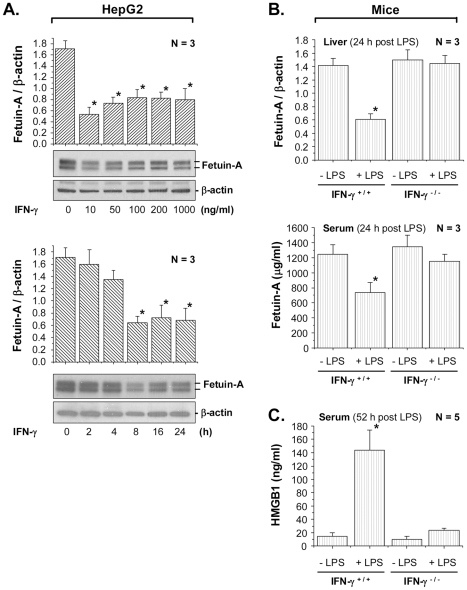
IFN-γ counter-regulates hepatic fetuin-A expression. **A**). *IFN-γ decreased fetuin-A expression levels in hepatocytes*. HepG2 cells were stimulated with IFN-γ for 16 h at different doses (**Top Panel**), or at 50 ng/ml for different time periods (**Bottom Panel**), and cellular fetuin-A/β-actin ratio was assessed by Western blotting analysis. **B, C**). *Disruption of IFN-γ expression rendered mice resistant to LPS-induced down-regulation of hepatic fetuin-A expression*. LPS (10 mg/kg) was administered into wild-type or IFN-γ-knockout Balb/C mice, liver and blood was harvested at 24 h (**Panel B**) or 52 h (**Panel C**) post endotoxemia to assess fetuin-A (**Panel B**) or HMGB1 (**Panel C**) levels by Western blotting analysis. Hepatic fetuin-A levels, as a ratio to β-actin, were expressed as mean ± SD of multiple independent experiments (N = 3–5). *, *P*<0.05 versus control (“-LPS”).

To understand the mechanisms underlying regulation of fetuin-A expression during LSI, we examined the impact of early cytokines on hepatic fetuin-A expression. Consistent with a previous report [26], an early cytokine, TNF-α (50–100 ng/ml), effectively inhibited fetuin-A expression in HepG2 cells (by >50–60%, data not shown). Moreover, another early cytokine, IFN-γ, at concentrations as low as 10–50 ng/ml, markedly inhibited hepatic fetuin-A expression (by 50–70%) in a time-dependent fashion (**Fig. 2A** **, bottom panel**). To confirm the role of IFN-γ in the regulation of fetuin-A expression, we investigated whether disruption of IFN-γ expression impairs endotoxin-mediated down-regulation of fetuin-A expression. The basal hepatic (**Fig. 2B** **, top panel**) and circulating (**Fig. 2B** **, bottom panel**) fetuin-A levels were not significantly different between IFN-γ-null and wild-type Balb/C mice. However, at the dose (10 mg/kg) that significantly reduced hepatic fetuin-A levels in wild-type mice (**Fig. 2B** **, top panel**), LPS did not significantly reduce neither hepatic (**Fig. 2B** **, top panel**) nor serum (**Fig. 2B** **, bottom panel**) fetuin-A levels in IFN-γ-knockout mice. These experimental data support an important role for IFN-γ in the counter-regulation of fetuin-A expression during an early stage of endotoxemia.

To understand the potential role for IFN-γ in the regulation of LPS-induced HMGB1 release, we determined whether disruption of IFN-γ expression abrogated LPS-induced systemic HMGB1 accumulation. Consistent with previous report [5], endotoxemia led to a significant increase in circulating HMGB1 levels in wild-type Balb/C mice (**Fig. 2C**). However, this endotoxin-induced systemic HMGB1 accumulation was almost completely abolished in IFN-γ-deficient mice (**Fig. 2C**), supporting an important role for IFN-γ in endotoxin-induced HMGB1 release.

### Disruption of fetuin-A expression renders animals more susceptible to endotoxemia and sepsis

**Figure 3:**
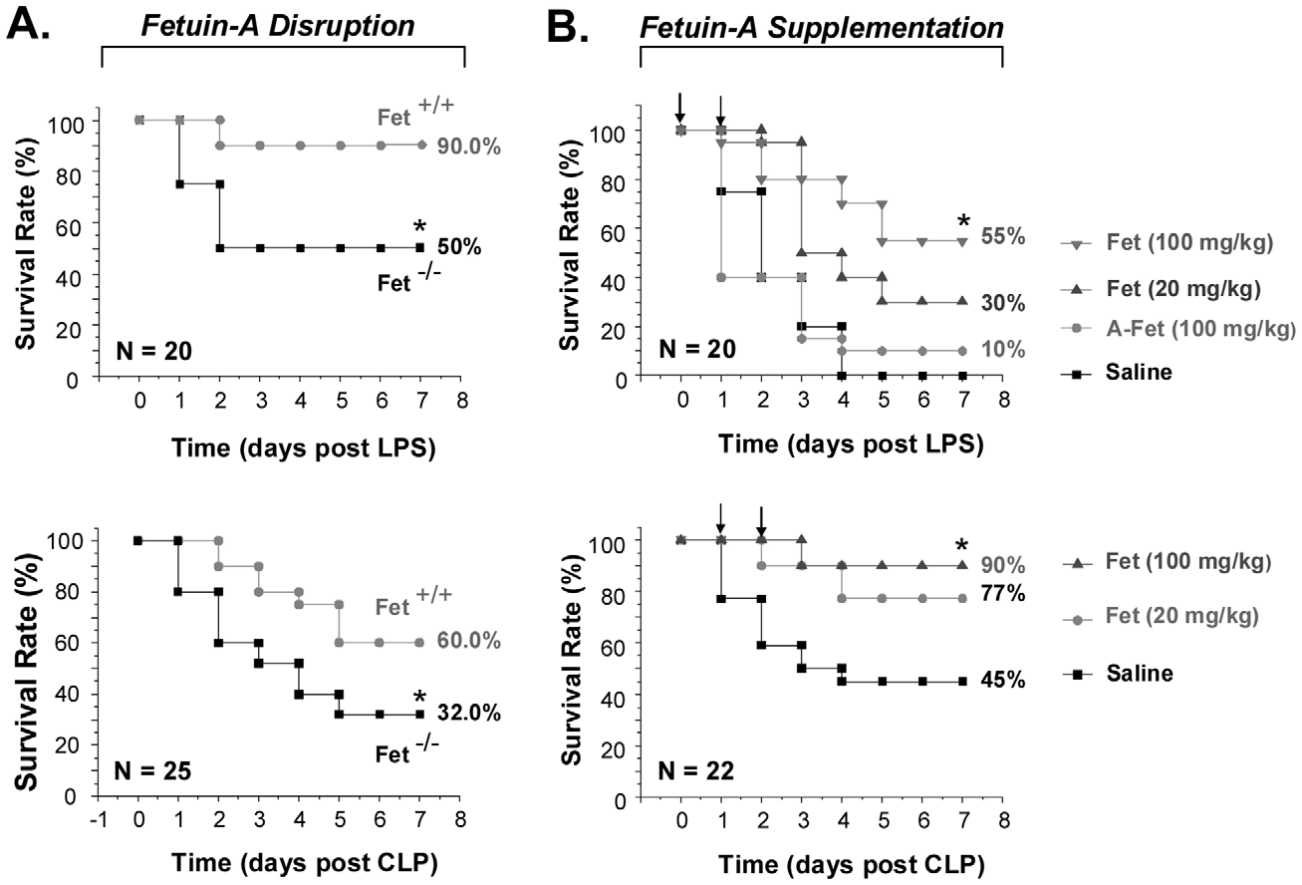
Distinct effects of fetuin-A depletion or supplementation on endotoxemic and septic lethality. ***A*** *). Disruption of fetuin-A expression rendered mice more susceptible to lethal endotoxemia and sepsis.* Sex-, body weight-, and genetic background-matched wild-type or fetuin-A-deficient (fet^−/−^) C57BL/6 mice (male, 27–29 g) were subjected to endotoxemia or sepsis, and animal survival was monitored. The Kaplan-Meier method was used to compare mortality rates between groups of two independent experiments with similar results. *, *p*<0.05 vs wild-type mice in endotoxemia (**Top Panel**) or sepsis (**Bottom Panel**). ***B*** *)*. *Supplementation of fetuin-A protected mice against lethal endotoxemia or sepsis.* Balb/C mice were challenged with lethal dose of endotoxin, and intraperitoneally administered with saline (0.2 ml/mouse), fetuin-A (“Fet”), or asialofetuin-A (“A-Fet”) at +0.5 and +24 h after endotoxemia. In separate experiments, Balb/C mice were subjected to sepsis (induced by CLP), and administered with fetuin-A at indicated doses at +24, and +48 h after CLP, and animal survival was monitored. *, *P*<0.05 versus saline.

To elucidate the role of fetuin-A in systemic inflammatory diseases, we determined the influence of fetuin-A disruption on endotoxemic and septic lethality. Sex- and body weight-matched wild-type or fetuin-A-knockout (KO) C57BL/6J mice were subjected to endotoxemia or sepsis, and animal survival rates were monitored. In an animal model of cerebral ischemia (local inflammation), there was no difference in susceptibility between sex- and body weight-matched (male, 27–30 g) wild-type and fetuin-A KO mice [43]. However, the animal survival rates were significantly lower in the fetuin-A KO mice as compared with wild-type C57BL/6J mice following endotoxemia (**Fig. 3A** **, top panel**) or sepsis (**Fig. 3A** **, bottom panel**). Consistently, disruption of fetuin-A expression led to significant elevation of serum HMGB1 levels at 48 h post endotoxemia (8226 ng/ml for Fet ^+/+^ mice, versus 181±45 ng/ml for Fet ^−/−^ mice; N = 10, *P*<0.05) or sepsis (125±46 ng/ml for Fet ^+/+^ mice, versus 271±34 ng/ml for Fet ^−/−^ mice; N = 12, *P*<0.05). These experimental data suggest a protective role for a liver-derived negative APP, fetuin-A, in systemic inflammatory diseases.

### Supplementation of fetuin-A conferred protection against lethal endotoxemia and sepsis

To confirm the role of fetuin-A in LSI, we examined its effects on animal survival in endotoxemia or sepsis. Repetitive administration of fetuin-A (20–100 mg/kg) promoted a dose-dependent protection against lethal endotoxemia (*P*<0.05, **Fig. 3B** **, top panel**). In contrast, administration of a control protein, asialofetuin-A, even at doses up to 100 mg/kg, did not significantly affect animal survival rates (**Fig. 3B** **, top panel**), suggesting a requirement for the presence of sialic acid in fetuin-A-mediated protection. In an animal model of sepsis, delayed administration of fetuin-A (20–100 mg/kg), beginning 24 h *after* the onset of sepsis and followed by an additional dose at 48 h post CLP, dose-dependently and significantly increased long-term animal survival rates from 45% to 90% (*P*<0.05, **Fig. 3B** **, bottom panel**). Although we did not observe any difference in the mortality rates between sex- and age-matched Balb/C and C57BL/6 mice in animal models of sepsis (induced by CLP), we noticed that wild-type C57BL/6J mice were somewhat less susceptible to endotoxemia than sex- and age-matched Balb/C mice (**Fig. 3A, 3B** **, top panels**).

**Figure 4:**
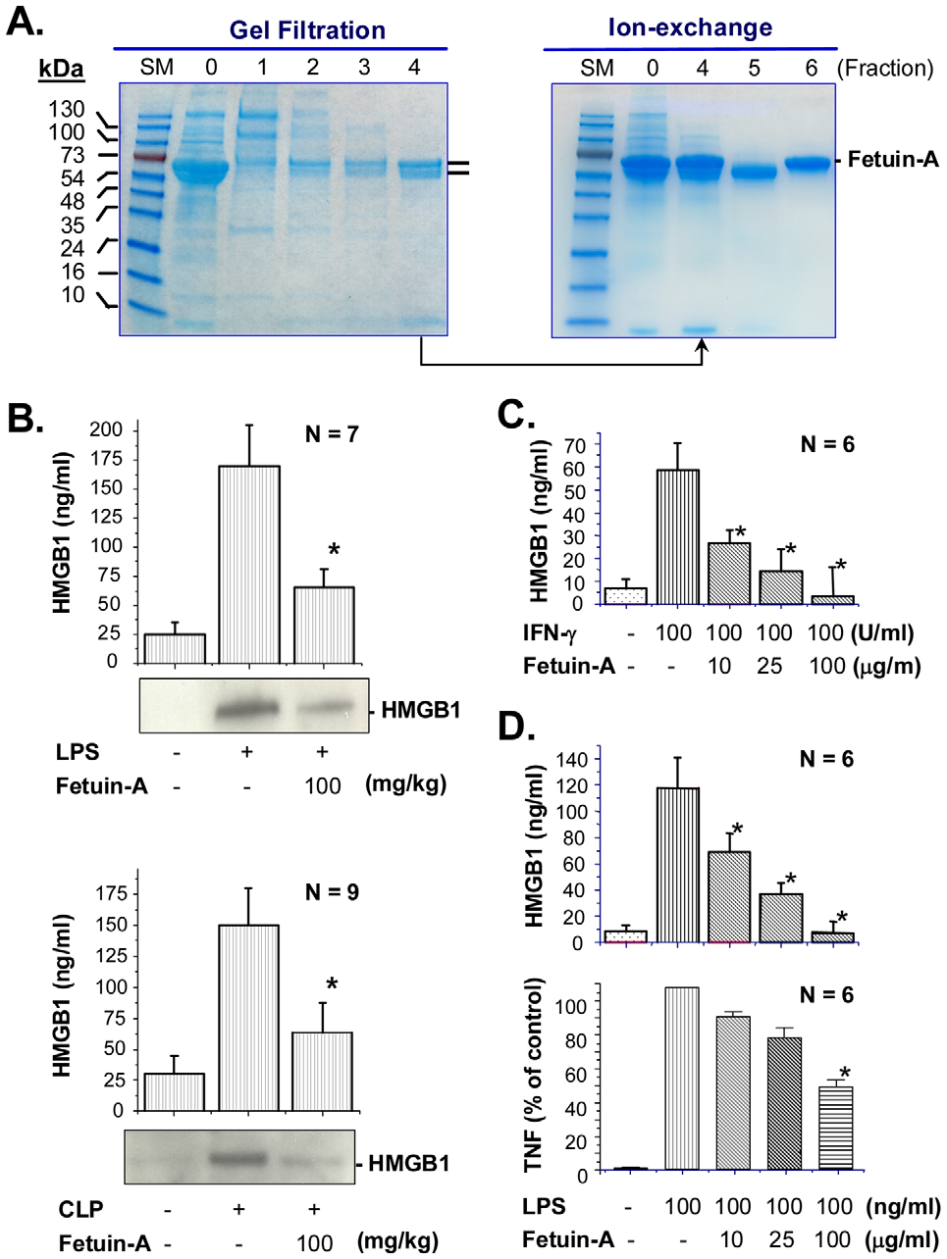
Highly purified fetuin-A inhibited HMGB1 release *in vivo* and *in vitro*. **A**). *Purification of fetuin-A by gel filtration and ion-exchange chromatography.* Bovine fetuin-A was obtained from Calbiochem, and further purified by gel filtration and ion-exchange chromatography. Lane “0”: crude fetuin-A from Calbiochem (Cat. #341506), Lane 1–4: consecutive gel filtration fractions. Gel filtration fraction #4 was further purified by ion-exchange chromatography. Lane 5–6: two major ion-exchange fractions containing a lower (Lane 5) and higher (Lane 6, “intact”) molecular weight protein. **B**). *Administration of fetuin-A decreased circulating HMGB1 levels in endotoxemia and sepsis.* Balb/C mice were subjected to lethal endotoxemia or sepsis, and treated with fetuin-A at +0.5, +24, and +48 h post endotoxemia, or +24 and +48 h post CLP. Blood were collected from normal, endotoxemic (52 h post LPS), or septic (52 h post CLP) mice, respectively. Serum HMGB1 levels were determined by Western blot analysis, and expressed as mean ± SD of two independent experiments in triplicates (N = 6). *, *P*<0.05 versus control “+LPS” or “+CLP” group. **C, D**). *Highly purified intact fetuin-A inhibited IFN-γ- or LPS-induced HMGB1 release in primary peritoneal macrophages*. Peritoneal macrophages were isolated form Balb/C mice, and stimulated with IFN-γ or LPS at indicated concentrations in the absence or presence of highly purified intact fetuin-A for 16 h. The culture medium was assayed for HMGB1 levels by Western blotting analysis (**Panel C, D**) and TNF-α levels by ELISA (**Panel D**), and expressed as Mean ± S.D. of two independent experiments in triplicates (N = 6). *, *P*<0.05 vs “+ IFN-γ” (**Panel C**) or “+ LPS” (**Panel D**).

Previously, it was shown that at high concentrations (e.g., 3500 µg/ml), fetuin-A (purity >98%, Sigma-Aldrich) itself slightly induced nitric oxide release in macrophage cultures [41], possibly because of chemical impurities contained in the fetuin-A preparation [41]. Indeed, SDS-PAGE analysis of “purified” fetuin-A preparation (Calbiochem, Cat. No. #341506) revealed multiple contaminating proteins that co-purified with the typical fetuin-A “doublet” bands on SDS-PAGE gels (**Fig. 4A**). This fetuin-A “doublet” may represent variable post-translation modifications (such as glycosylation and/or phosphorylation), and could be separated from other contaminating proteins by gel filtration (**Fig. 4A, left panel**), and from each other by ion-exchange chromatography (**Fig. 4A, right panel**). Even at lower doses (10 mg/kg), delayed administration of this highly purified fetuin-A significantly increased animal survival rates from 45% to 90% (N = 22 mice/group, *P*<0.05). Taken together, these experimental data suggest that fetuin-A is protective against lethal systemic inflammatory diseases.

To gain insight into its protective mechanism, we evaluated the effects of fetuin-A on systemic accumulation of HMGB1 during a late stage of endotoxemia and sepsis. Administration of fetuin-A significantly reduced endotoxemia- or sepsis-induced increase of circulating HMGB1 levels at 52 h post endotoxemia (**Fig. 4B** **, top panel**) or sepsis (**Fig. 4B** **, bottom panel**), suggesting that fetuin-A confers protection by inhibiting systemic accumulation of late proinflammatory mediator of these diseases.

### Highly purified fetuin-A inhibited active HMGB1 release in macrophage cultures

To elucidate the mechanisms underlying fetuin-A-mediated suppression of systemic HMGB1 accumulation *in vivo*, we examined the effects of fetuin-A on IFN-γ- and endotoxin-induced HMGB1 release in macrophage cultures. Following extensive purification by gel filtration (**Fig. 4A, left panel**) and ion-exchange chromatography (**Fig. 4A, right panel**), the intact fetuin-A was capable of inhibiting IFN-γ- (**Fig. 4C**) and LPS-induced HMGB1 release (**Fig. 4D** **, top panel**). Even at the concentrations (e.g., 100 µg/ml) that almost completely abrogated LPS-induced HMGB1 release, fetuin-A only partly inhibited LPS-induced TNF-α secretion (**Fig. 4D**, **bottom panel**), suggesting that highly purified fetuin-A is an effective negative regulator of HMGB1 release.

### Fetuin-A did not inhibit endotoxin-induced autophagy, but reduced cytoplasmic HMGB1 levels

**Figure 5:**
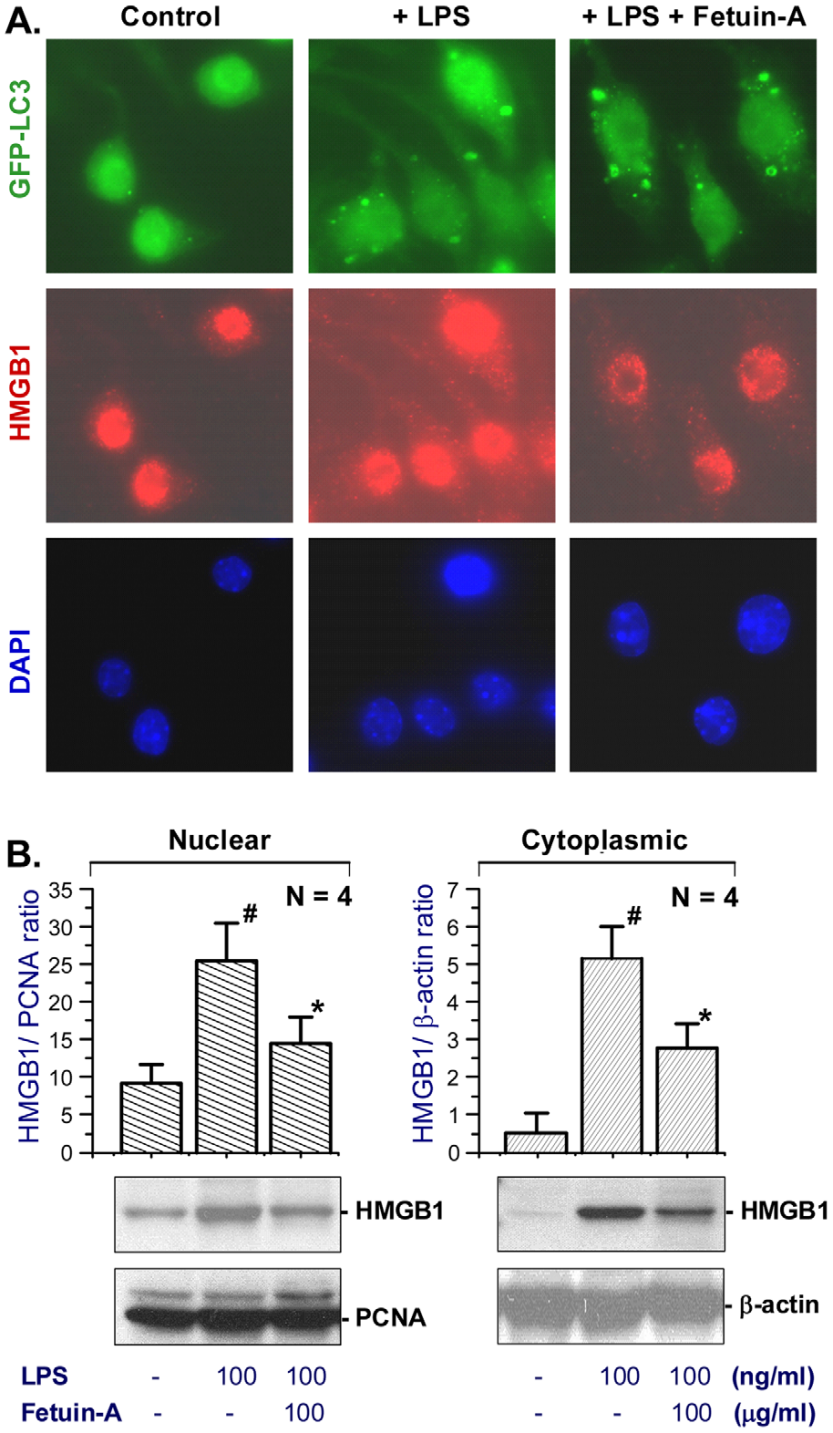
Fetuin-A did not affect LPS-induced formation of LC3-containing cytoplasmic vesicles, but attenuated cytoplasmic HMGB1 levels. A). *Fetuin-A did not affect LPS-induced formation of autophagosomes, but reduced cytoplasmic HMGB1 staining*. GFP-LC3 transfected macrophages were stimulated with LPS (200 ng/ml) in the absence or presence of fetuin-A, and immunostained with HMGB1-specific antibodies. Note that HMGB1 was predominantly localized in the nuclear region of un-stimulated macrophages (control) (*left panels*), but in both cytoplasmic and nuclear regions of LPS-stimulated macrophages (*middle panels*). Fetuin-A reduced cytoplasmic HMGB1 staining in LPS-stimulated macrophages (LPS + fetuin-A). Images are representative of three independent experiments with similar results. B). *Fetuin-A reduced cytoplasmic and nuclear HMGB1 levels in LPS-stimulated macrophages.* Thioglycollate-elicited peritoneal murine macrophages were stimulated with LPS in the absence or presence of fetuin-A (100 µg/ml) for 16 h, and cytoplasmic nuclear fractions were isolated, and assayed for levels of HMGB1 with reference to a nuclear (PCNA) or cytoplasmic (β-actin) markers. Blots are representative of two independent experiments with similar results. Bar graphs were mean ± SD of two independent experiments in duplicates (N = 4). ^#^, *P*<0.05 vs negative control “- LPS”; *, *P*<0.05 vs positive control “+ LPS”.

A previous study has implicated a potential role for autophagy in the regulation of endotoxin-induced HMGB1 release, because an HMGB1 inhibitor, quercetin, simultaneously inhibits LPS-induced formation of LC3-containing cytoplasmic vesicles (autophagosome) and HMGB1 release [44]. To elucidate mechanisms underlying fetuin-A-mediated suppression of HMGB1 release, we determined whether fetuin-A affects LPS-induced formation of LC3-containing cytoplasmic vesicles (autophagosomes). Consistent with a previous report [45], LPS induced the formation of LC3-containing cytoplasmic vesicles (autophagosomes) in GFP-LC3-transfected macrophage cultures (**Fig. 5A**). In contrast to quercetin, however, fetuin-A failed to inhibit LPS-induced formation of LC3-containing punctuate structures in macrophage cultures (**Fig. 5A** **, top panels**). By itself, fetuin-A stimulated the formation of LC3-containing punctate structures (data not shown), suggesting a possibility that fetuin-A inhibits HMGB1 release potentially by stimulating its autophagic degradation.

In addition, we determined whether fetuin-A affects cytoplasmic HMGB1 levels in endotoxin-stimulated macrophages. Quiescent macrophages constitutively expressed HMGB1 and maintained an intracellular “pool” of HMGB1 predominantly in the nucleus (**Fig. 5A, left panel** **s**). At 16 h post LPS stimulation, marked HMGB1 staining was observed in cytoplasmic vesicles (**Fig. 5A** **, middle panels**). However, fetuin-A markedly reduced HMGB1 staining in cytoplasmic regions (**Fig. 5A, right panel** **s**), suggesting a possibility that fetuin-A attenuates HMGB1 release by reducing its cytoplasmic levels. To further test this possibility, cytoplasmic and nuclear fractions were isolated from primary macrophages, and immunoblotted with antibodies specific for HMGB1, PCNA (a nuclear protein), or β-actin (a cytoplasmic protein), respectively. In murine macrophage-like RAW 264.7 cells, LPS merely induced cytoplasmic HMGB1 translocation and release, but did not increase nuclear (or total cellular) HMGB1 levels [5]. In primary murine peritoneal macrophages, however, LPS significantly elevated HMGB1 levels in both cytoplasmic and nuclear fractions (**Fig. 5B**). At the concentrations (100 µg/ml) that significantly inhibited LPS-induced HMGB1 release, fetuin-A significantly reduced both nuclear and cytoplasmic HMGB1 levels (**Fig. 5B**).

## Discussion

In response to infection or injury, the liver re-prioritizes the synthesis and systemic release of many APPs. One hepatic protein, fetuin-A, has previously been suggested either as a negative or positive APP following infection- or injury-elicited inflammation [29]–[31]. In the present study, we found that circulating fetuin-A levels were time-dependently decreased during endotoxemia and sepsis, supporting the notion that fetuin-A functions as a negative APP during LSI.

**Figure 6:**
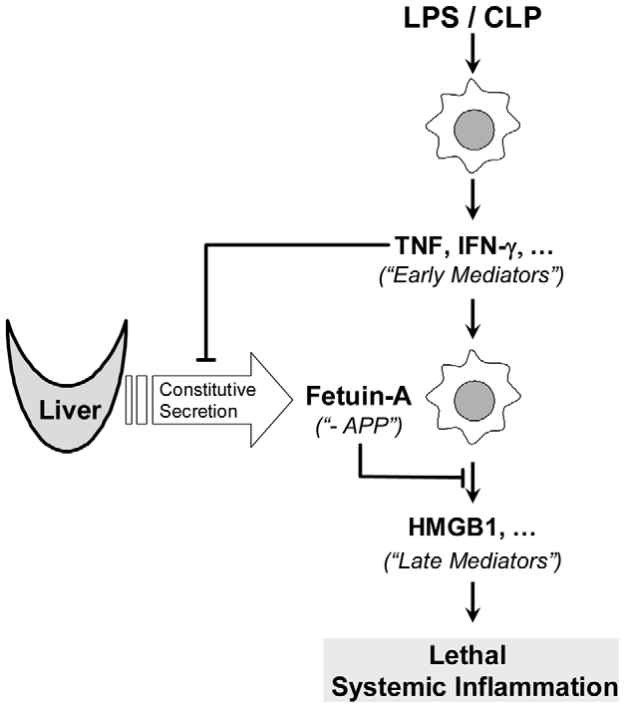
Hypothetical role of fetuin-A in lethal systemic inflammation. Fetuin-A is predominantly synthesized in, and constitutively secreted by the liver to maintain abundant basal circulating levels (e.g., 1100–1350 µg/ml in mice). In response to lethal endotoxemia (LPS) or sepsis (CLP), innate immune cells (such as macrophages) sequentially release early (e.g., TNF-α and IFN-γ) and late (e.g., HMGB1) proinflammatory mediators. These early proinflammatory cytokines (e.g., TNF-α, IFN-γ and perhaps others) participate in the down-regulation of hepatic fetuin-A expression, allowing propagation of a rigorous inflammatory response manifested by excess accumulation of late proinflammatory mediators (such as HMGB1). On the other hand, fetuin-A functions as a negative regulator of the innate immune response by inhibiting LPS- or IFN-γ-induced HMGB1 release in macrophages.

During endotoxemia or sepsis, multiple early cytokines (such as TNF-α and IFN-γ) are responsible for counter-regulating hepatic fetuin-A expression, thereby reducing circulating fetuin-A levels (**Fig. 6**). Indeed, disruption of IFN-γ expression impaired endotoxin-induced suppression of hepatic fetuin-A expression *in vivo*. It is thus plausible that IFN-γ, a proinflammatory cytokine predominantly derived from spleen [46], contributes to lethal endotoxemia [47], [48] or sepsis [49] partly by stimulating HMGB1 release [50] and partly by inhibiting hepatic fetuin-A expression.

A previously under-appreciated protective role for fetuin-A in LSI has been suggested in the present study. First, the disruption of fetuin-A expression rendered mice more susceptible to endotoxemia or sepsis. Second, repetitive administration of fetuin-A conferred a dose-dependent protection against these systemic inflammatory diseases. In light of our observation that administration of fetuin-A markedly reduced circulating levels of HMGB1, but not TNF-α (data not shown), we propose that fetuin-A confers protection against lethal endotoxemia and sepsis partly by inhibiting late mediators of these diseases. Nevertheless, the current study can not exclude other alternative mechanisms by which fetuin-A confers these protective effects. For instance, fetuin-A may be capable of binding bacteria [51], [52], thereby affecting macrophage-mediated pathogen elimination. Furthermore, fetuin-A may facilitate macrophages-mediated ingestion and elimination of apoptotic neutrophils [53], [54], thereby preventing secondary necrosis and passive leakage of injurious molecules (e.g., proteases, reactive oxygen species, and HMGB1) [55].

*In vitro*, highly purified intact fetuin-A effectively inhibited IFN-γ- and endotoxin-induced HMGB1 release in macrophage cultures. These inhibitory effects were concentration-dependent, and required the presence of sialic acid in the intact fetuin-A. Although it is difficult to correlate the concentration-effect relationship of fetuin-A *in vitro* and *in vivo*, a single injection of fetuin-A at 100 mg/kg could theoretically produce a minimal tissue level of 100 µg/ml fetuin-A (assuming even distribution in all tissues including bone, muscle, blood, and others). It is thus possible that the fetuin-A-mediated inhibition of IFN-γ- or LPS-induced HMGB1 release *in vitro* partly accounts for the observed inhibition of serum HMGB1 levels *in vivo*. We propose that endogenous fetuin-A functions as a negative regulator of HMGB1 release during lethal systemic inflammation. First, the time-dependent decrease of circulating fetuin-A levels is accompanied by parallel but contrary changes - a time-dependent increase - of circulating HMGB1 levels in animal model of endotoxemia [5] or sepsis [17]. Second, disruption of fetuin-A expression led to significant elevation of serum HMGB1 levels during endotoxemia and sepsis. Lastly, supplementation of fetuin-A resulted in significant reduction of circulating HMGB1 levels during endotoxemia and sepsis.

The mechanisms underlying fetuin-A-mediated suppression of HMGB1 release may be complex. For instance, fetuin-A may attenuate systemic HMGB1 accumulation indirectly by facilitating macrophage-mediated phagocytotic elimination of apoptotic cells [54]. This is relevant because prolonged accumulation of apoptotic cells may allow these cells to enter secondary necrosis, leading to rapid HMGB1 leakage. In addition, at the concentrations (100 µg/ml) that significantly inhibited LPS-induced HMGB1 release, fetuin-A stimulated the formation of LC3-containing punctuate structures (likely autophagosomes), and impaired LPS-induced elevation of both cytoplasmic and nuclear HMGB1 levels. At present, it is not yet known whether fetuin-A reduces cytoplasmic HMGB1 levels by transcriptionally down-regulating HMGB1 expression, or stimulating its degradation in an autophagy-dependent fashion. Nevertheless, the fetuin-A-mediated reduction of cytoplasmic HMGB1 levels may underlie its inhibition of endotoxin-induced HMGB1 release in macrophage cultures.

In summary, we demonstrated that circulating fetuin-A levels was time-dependently reduced during lethal endotoxemia and sepsis, supporting the notion that fetuin-A functions as a negative APP during LSI. The temporal decrease of circulating fetuin-A levels ensures a rigorous innate immune response manifested by excessive accumulation of early (e.g., IFN-γ) and late (e.g., HMGB1) proinflammatory mediators. Supplementation with exogenous fetuin-A could tilt the balance towards inhibiting active HMGB1 release (**Fig. 6**). Thus, fetuin-A occupies an important protective role against LSI by counter-regulating systemic accumulation of late mediators (e.g., HMGB1).

## Materials and Methods

### Animal models of lethal endotoxemia and sepsis

This study was approved and performed in accordance with the guidelines for the care and use of laboratory animals at the Feinstein Institute for Medical Research. Sex-, weight-, and genetic background-matched (male, 23–25 g) wild-type and fetuin-A-deficient C57BL/6J mice were obtained from the Jackson Laboratory (Bar Harbor, ME, USA) and Dr. Willi Jahnen-Dechent's laboratory, respectively. Wild-type Balb/C mice and IFN-γ-deficient Balb/C mice were also obtained from the Jackson Laboratory.

Endotoxemia was induced by intraperitoneal injection of endotoxin (lipopolysaccharide, LPS, *E. coli 0111:B4*, Sigma-Aldrich, 10 mg/kg) as previously described [5]. As a clinically relevant model, sepsis was induced by cecal ligation and puncture (CLP) as previously described [20], [22], [56]. Purified fetuin-A (Calbiochem, Cat. No. #341506) or asialofetuin-A (sialic acid residues of fetuin-A removed by neuraminidase) were administered intraperitoneally into mice, and animal survival rates were monitored. In parallel experiments, mice were euthanized at indicated time points to collect blood (by cardiac puncture) or liver tissue, to measure HMGB1 or fetuin-A by Western blotting analysis.

### Cell culture

Murine macrophage-like RAW 264.7 cells and human hepatocyte HepG2 cells were obtained from the American Type Culture Collection (ATCC, Rockville, MD). GFP-LC3-transfected RAW 264.7 cells were established as previously described [45]. Primary peritoneal macrophages were isolated from Balb/C mice (male, 7–8 weeks, 20–25 grams) at 3 days after intraperitoneal injection of 2 ml thioglycollate broth (4%) as previously described [20], [22], [50], [57]. Murine macrophages were pre-cultured in DMEM medium (Gibco BRL, Grand Island, NY) supplemented with 10% fetal bovine serum (FBS), 2 mmol/L glutamine, and 1% penicillin. Adherent macrophages or HepG2 cells were gently washed with, and cultured in, OPTI-MEM I medium 2 h before stimulation with LPS, or IFN-γ (Sigma-Aldrich, Cat. No. 14777, Louis, MO), in the absence or presence of fetuin-A at indicated concentrations. At indicated time points after stimulation, intracellular or extracellular levels of HMGB1, TNF-α, or fetuin-A, were determined by Western blotting analysis or ELISA as previously described [20], [50].

### Purification of intact fetuin-A

Gel filtration chromatography was performed using a HiPrep^TM^ 26/60 Sephacryl S-100 high resolution column. Sample was eluted by 1x PBS at a flow rate of 1.0 ml/min, and gel filtration fractions were subjected to protein analysis by SDS-PAGE gel electrophoresis. The filtration fraction enriched in fetuin-A protein was further purified by ion-exchange chromatography. Briefly, gel filtration fraction was loaded onto a 5-ml HiTrap^TM^ SP HP column pre-equilibrated in buffer A (50 mM NaAc, pH 7.5), and the column was washed with 5% Buffer B (1.0 M NaCl in 50 mM NaAc) until the A_280_ dropped below 1% of its maximum. Proteins bound to the column were eluted by a linear gradient of 0–20% Buffer B over 40 min at a flow rate of 2.5 ml/min, and ion-exchange fractions were subjected to purity analysis by SDS-PAGE gel electrophoresis.

### Western blotting analysis

Following SDS-PAGE electrophoresis, proteins were hybridized with specific primary antibodies. Rat HMGB1-specific and bovine fetuin-A-specific polyclonal antibodies were generated in rabbits as previously described [5], [43]. Human fetuin-A-specific polyclonal antibodies were obtained from Santa Cruz (Cat. No. sc-9663). Monoclonal antibodies against β-actin were obtained from Abcam (Cat. No. mAbcam 8226). After incubation with the alkaline phosphotase-conjugated secondary antibodies, the signal was detected with the colormetric alkaline phosphatase assay kit (Bio-Rad Laboratories). The relative band intensity was quantified by using the NIH Image 1.59 software to determine fetuin-A levels with reference to β-actin, or HMGB1 levels with reference to standard curves generated with purified HMGB1 as previously described [22], [43].

### TNF-α ELISA

TNF-α levels were determined using ELISA kits (Catalog no. MTA00, R & D Systems, Minneapolis, MN) with reference to standard curves of purified recombinant TNF-α at various dilutions as previously described [20], [22].

#### Visualization of LC3-containing cytoplasmic vesicles (autophagosomes)

Autophagy, literally meaning “self-eating”, refers to an evolutionarily conserved process for degrading organelles and cytoplasmic macromolecules. It begins with the formation of double-membraned structures called phagophores, which elongate and engulf portions of cytoplasm to form autophagosomes. The basic principle of autophagy assays is to measure the transfer of a soluble, membrane-impermeant LC3 protein from cytosol to autophagic vesicles (autophagosomes). Murine macrophage-like RAW 264.7 cells stably transfected with GFP-LC3 were stimulated with LPS in the absence or presence of fetuin-A for 16 h, and cells were examined for the presence GFP-LC3-II punctate structures under fluorescence microscope as previously described [45].

### Fluorescence Immunostaining

Macrophage cultures were fixed with 2% formalin (10 min), and permeabilized with 0.1% Triton X-100 (in PBS, 1 min, room temperature). After extensive washing with 1x PBS, cells were incubated sequentially with antigen-affinity-purified rabbit anti-HMGB1 antibodies or anti-fetuin-A polyclonal Abs, donkey anti-rabbit secondary antibodies conjugated with red Alexa fluor 594 (Invitrogen, Cat # 404239, Eugene, OR), and Vectashield mounting medium with DAPI (Vector, Cat #1200, Burlingame, CA). Images were captured using a fluorescence microscope (Carl Zeiss Microimaging) as previously described [43]. Alternatively, localization of HMGB1 was examined by a cell fractionation/Western blotting technique as previously described [20]. After fractionation, the protein content of different fractions was determined by a Bradford method, and each fraction was assayed for levels of various protein by Western blotting analysis using primary antibodies specific for HMGB1, a cytoplasmic protein (β-actin, Santa Cruz Biotechnology), and a nuclear protein (PCNA, BD Biosciences).

### Statistical Analysis

Data are expressed as mean ± SD of at least 2–3 independent experiments (n = 2–3). One-way ANOVA was used for comparison among all different groups. When the ANOVA was significant, post-hoc testing of differences between groups was performed using Tukey's test. A *P* value <0.05 was considered statistically significant. The Kaplan-Meier method was used to compare the differences in mortality rates between groups. A *P* value <0.05 was considered statistically significant.
